# Comparison of medical image interpretation time between conventional and automated methods of breast ultrasound

**DOI:** 10.61622/rbgo/2024AO15

**Published:** 2024-03-15

**Authors:** Katyane Larissa Alves, Ruffo Freitas, Régis Resende Paulinelli, Marcus Nascimento Borges

**Affiliations:** 1 Universidade Federal de Goiás Goiânia GO Brazil Universidade Federal de Goiás, Goiânia, GO, Brazil.

**Keywords:** Breast ultrasound, Diagnostic imaging, Breast neoplasms, Three-dimensional imaging

## Abstract

**Objective:**

To compare the medical image interpretation's time between the conventional and automated methods of breast ultrasound in patients with breast lesions. Secondarily, to evaluate the agreement between the two methods and interobservers.

**Methods:**

This is a cross-sectional study with prospective data collection. The agreement's degrees were established in relation to the breast lesions's ultrasound descriptors. To determine the accuracy of each method, a biopsy of suspicious lesions was performed, considering the histopathological result as the diagnostic gold standard.

**Results:**

We evaluated 27 women. Conventional ultrasound used an average medical time of 10.77 minutes (± 2.55) greater than the average of 7.38 minutes (± 2.06) for automated ultrasound (p<0.001). The degrees of agreement between the methods ranged from 0.75 to 0.95 for researcher 1 and from 0.71 to 0.98 for researcher 2. Among the researchers, the degrees of agreement were between 0.63 and 1 for automated ultrasound and between 0.68 and 1 for conventional ultrasound. The area of the ROC curve for the conventional method was 0.67 (p=0.003) for researcher 1 and 0.72 (p<0.001) for researcher 2. The area of the ROC curve for the automated method was 0. 69 (p=0.001) for researcher 1 and 0.78 (p<0.001) for researcher 2.

**Conclusion:**

We observed less time devoted by the physician to automated ultrasound compared to conventional ultrasound, maintaining accuracy. There was substantial or strong to perfect interobserver agreement and substantial or strong to almost perfect agreement between the methods.

## Introduction

In clinical practice, breast ultrasound plays an important role in the investigation of mammographic and clinical findings, helping to differentiate between cysts and solid nodules and in the characterization of solid nodules as probably benign or suspicious for malignancy. It may also show some additional lesions, possibly not identified on mammography and/or physical examination. This is especially true in women with a dense fibroglandular tissue pattern, which reduces the sensitivity of mammography.^(1)^

However, conventional breast ultrasound has some limitations, such as the considerable medical time required to obtain and interpret the images. The inter-observer variability and the increase in the number of false-positives, generating increased costs, add to this and make the applicability of the screening method remain controversial.^(2)^

Automated breast ultrasound was developed and initially used in the context of screening women with dense breasts, as a complement to mammography. With a transducer larger than the conventional one, coupled to a mechanical arm, the automated ultrasound device performs an automatic and standardized scan of the entire breast. The images obtained are transferred to a workstation where they are available for medical interpretation, allowing, for example, double reading.^(3)^ The systematization of image acquisition improves reproducibility, reducing interobserver variability.^(4)^

Initially, the objective was to automate the method to optimize the medical time for evaluating the ultrasound images. With the transfer of image acquisition time to a radiology technician with specific training, there is the possibility of using the method on a large scale.^(5,6)^ In the diagnostic context, although its use in patients with suspicious lesions has already been the subject of some studies, its indication still remains uncertain.^(3,7,8)^

Thus, we aimed to compare the time dedicated by the physician to the images’ interpretation obtained by automated breast ultrasound (ABUS) with the time spent by the physician performing conventional breast ultrasound (reported as HHUS= hand held ultrasound) in patients with breast lesions. Secondarily, we also aimed to evaluate the agreement between the two methods and interobservers regarding the American College of Radiology Breast Image Reporting and Data System (ACR BI-RADS^®^) classification of breast lesions and regarding the echographic descriptors of the lesions submitted to biopsy for diagnostic investigation.

## Methods

This is a cross-sectional study with prospective data collection. The study population consisted of patients from the Unified Health System treated at the ultrasound-guided breast biopsy clinic of the Mastology Program at *Hospital das Clínicas* of the Federal University of Goiás, carried out at Advanced Centre Breast Diagnosis in Goiânia-GO. The patients who agreed to participate were included in the study, after being instructed about the research and having signed the free and informed consent form.

We included patients aged over 18 years, with breast lesions (categorized as BI-RADS 3, 4 or 5 by breast imaging, according to the standard reports of the American College of Radiology (ACR BI-RADS^®^) referred to mastology's service to perform a core-needle breast biopsy (core-biopsy). Under this system, the suspicion's degree differs according to the category, being 0-2% for category 3, between 2-10% for category 4A, between 10-50% for category 4B, between 50-95% for category 4C and ≥ 95% for category 5.^(9)^

We excluded patients under 18 years of age and men.

The sample size estimate was obtained from the interpretation time of the automated breast ultrasound images compared to the time taken by the physician to perform the conventional breast ultrasound. We used the mean and standard deviation of each times to determine the size of the sample effect using the G. Power^®^ 3.1 software.

The average time for automated breast ultrasound was 7.38 minutes (SD±2.06) and for conventional breast ultrasound was 10.77 minutes (SD±2.55). The confidence interval adopted was 0.95, the sampling error 0.05 and the sample power 80%. From these parameters, a sample effect size of 1.51 was obtained. In this way, a minimum estimate of 26 women in the sample was verified.

In order to carry out this research, the physicians and the radiology technician received specific training standardized by GE Healthcare in handling the Invenia ABUS device and interpreting, in the physicians’ case, the data obtained by automated breast ultrasound.

Each study participant underwent automated and conventional ultrasound of the breasts on the same date and period in which they attended for a breast biopsy guided by conventional ultrasound. Two mastologists were observers of this study. Each of the patients included underwent the automated examination with the radiology technique and, soon after, the conventional examination with each of the two physicians, independently. Next, a biopsy of the breast lesions was performed with indication for diagnostic investigation. The automated data obtained were analyzed on another date by each of the observing physicians, also independently.

Medical time was considered as the time between the beginning of gel application and the end of breast evaluation/end of transducer contact with the patient's breast in conventional ultrasound. For automated ultrasonography, medical time was considered as the time between the beginning of the opening of the images in the workstation and the end of the evaluation of these images. To mark time, the same digital stopwatch was used throughout the research data collection, handled by the main researcher or by the second observer.

The equipment used to perform the conventional ultrasound was a LOGIQ S8 Xdclear 2.0 with oLED (Brazilian GE Healthcare). For automated ultrasound, the Invenia ABUS (GE Healthcare; Sunnyvale, CA, USA) was used.

Data were analyzed using the statistical package SPSS (Statistical Package for Social Sciences) version 26, adopting a significance level of 5% (p < 0.05). The characterization of the patients’ sociodemographic and clinical profile was performed using descriptive statistics: median, mean, standard deviation, minimum and maximum for continuous variables. For categorical variables, absolute frequency and relative frequency were used.

The choice of using parametric or non-parametric tests was made after performing the Kolmogorov-Smirnov normality test. The comparison of the time taken to interpret data from the automated breast ultrasound with the time taken by the physician to perform the conventional breast ultrasound was performed using the parametric paired t test, as it is a variable with normal distribution.

The analysis of agreement between both methods and interobservers was performed using the Kappa index and Kendall's Tau-b-correlation coefficient. The following degrees of agreement are assigned:

- 0 - 0,2: weak
- 0,21 - 0,4: reasonable
- 0,41 – 0,6: moderate
- 0,61 – 0,8: strong or substantial
- 0,81 – 1: almost perfect
- 1: perfect

The agreement's degrees between the methods and interobservers were established in relation to the BI-RADS^®^ classification of breast lesions and in relation to the echographic descriptors of the lesions submitted to biopsy for diagnostic investigation. For the BI-RADS^®^ classification, lesions were grouped into benign when they were categorized as BI-RADS^®^ 1, 2 or 3 and suspicious when they were categorized as BI-RADS^®^ 4 (A, B or C) or 5.

Analysis of the ROC curve made it possible to assess the sensitivity, specificity and accuracy of each method by comparing the BI-RADS^®^ classification of the breast lesion with the histopathological result defined as the diagnostic gold standard.

The evaluation of the indicators related to the accuracy of each method was performed by using the Galen and Gambino's method (1975).^(10)^

The study was approved by the Research Ethics Committee of the Hospital das Clínicas, Federal University of Goiás, opinion n°4.983.602.

## Results

We evaluated 27 women, whose demographic and clinical profile are described in table 1.

**Table 1:** Characterization of the demographic and clinical profile of the women participating in the research

		Mean ± SD	n(%)
Age years)		48,78 ± 11,85	-
Weight (Kg)		70,79 ± 16,12	-
Height (m)		1,60 ± 0,07	-
BMI kg m^2^		27,60 ± 5,76	-
Age group			
	26 a 59	-	22(81,5)
	60 a 80	-	5(18,5)
BMI			
	< 25	-	10(37,0)
	≥ 25	-	17(63,0)
Menopausal state			
	pre menopause	-	16(59,3)
	post menopause	-	11(40,7)
Family history of breast cancer			
	No	-	22(81,5)
	Yes	-	5(18,5)
Personal history of breast cancer			
	No	-	25(92,6)
	Yes	-	2(7,4)
Breast surgery			
	No	-	25(92,6)
	Yes	-	2(7,4)
Breast reconstruction			
	No	-	26(96,3)
	Yes	-	1(3,7)
Breast implant or expander			
	No	-	27(100,0)
	Yes	-	0(0,0)
Breast radiotherapy			
	No	-	25(92,6)
	Yes	-	2(7,4)
Breast asymmetry			
	No	-	25(92,6)
	Yes	-	2(7,4)
Breast size			
	Small	-	3(11,1)
	Averages	-	10(37,0)
	Large	-	14(51,9)

n - absolute frequency; % - relative frequency; SD= standard deviation

**Figure 1:**
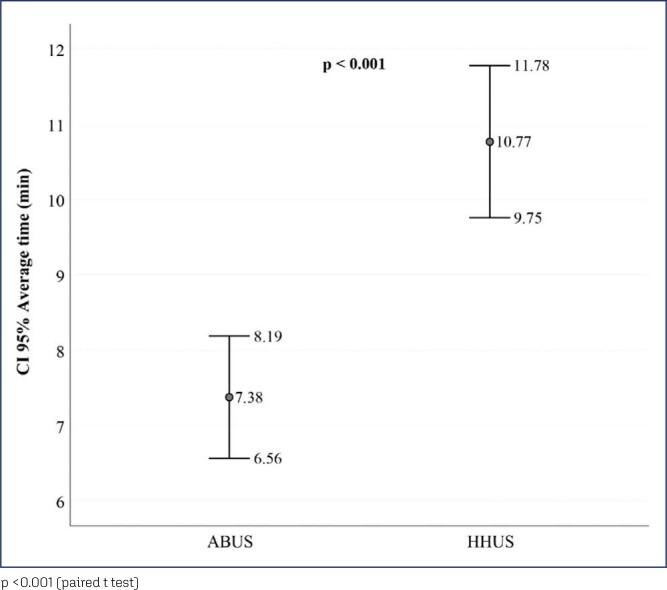
Error bar graph showing the result of comparing medical time between automated (ABUS) and conventional method (HHUS)

Twenty-four biopsies were performed and in three cases there was no need for the procedure after echographic reassessment and conclusion that there was no suspicious lesion to be investigated. The histopathological diagnosis of malignancy was observed in six cases (22.2% of the sample), predominantly invasive ductal carcinoma not otherwise specified, four cases. Among the benign histologies, fibroadenomas and benign breast tissue predominated, seven cases and six cases, respectively. The cases categorized as BI-RADS^®^ 1 and 2 corresponded to the 3 cases in which the biopsy was not performed. The 5 cases categorized as BI-RADS^®^ 3 in both methods and by both investigators resulted in benign histologies, fibroadenomas, one of them juvenile, and benign breast tissues. Of the 19 cases classified as BI-RADS^®^ 4 (A,B or C) and 5 only 6 corresponded to malignant histologies, 5 of them BI-RADS^®^ 4B or 4C or 5. In a single case of malignancy the BI-RADS^®^ classification had been 4A for both methods in investigator 1's assessment and in investigator 2's assessment it had been BI-RADS^®^ 4A for the conventional method and 4B for automated. Data normality was verified using the Kolmogorov-Smirnov test, observing normal distribution for the following variables: time, age, weight, height, BMI (body mass index), distance from the lesion to the mammary papilla and lesion size. Thus, it was possible to use the paired T parametric test to compare the time used by each of the researchers for the medical images’ interpretation in each method (Figure 1).

Conventional breast ultrasound used an average medical time of 10.77 minutes (SD ± 2.55) greater than the average of 7.38 minutes (SD ± 2.06) for automated breast ultrasound (p<0.001, paired t-test). Also noteworthy is the average time of 23.3 minutes (SD±3.64) used by the radiology technician to position the patient and obtain automated images. The agreement regarding the BI-RADS^®^ classification of breast lesions and regarding the echographic descriptors of the lesions submitted to biopsy is shown in table 2. An almost perfect agreement between the two methods for researcher 1 is observed in all variables, except for orientation (parallel and not-parallel) of the lesion in relation to the skin, which showed substantial or strong agreement. For researcher 2, the variables echogenicity, margins and posterior acoustic changes showed almost perfect agreement and the other variables (BI-RADS^®^ classification, shape and orientation) had substantial or strong agreement between the two methods.

**Table 2:** Characterization and agreement analysis between the methods regarding the BI-RADS^®^ classification of breast lesions and regarding the echographic descriptors of the lesions submitted to biopsy, with data from both researchers

		P1 n(%)		K	P2 n(%)		K
		ABUS	HHUS		ABUS	HHUS	
BI-RADS^^®^^							
	Benign	9(33,3)	9(33,3)	0,92*	13(48,1)	11(40,7)	0,70*
	Suspect	18(66,7)	18(66,7)		14(51,9)	16(59,3)	
Echogenicity							
	Hypoechoic	10(41,7)	9(37,5)	0,90**	10(41,7)	11(45,8)	0,87**
	Isoechoic	0(0,0)	0(0,0)		1(4,2)	0(0,0)	
	Heterogeneous	8(33,3)	9(37,5)		8(33,3)	8(33,3)	
	> 1 pattern	3(12,5)	3(12,5)		1(4,2)	1(4,2)	
	Solid-cystic	3(12,5)	3(12,5)		4(16,7)	4(16,7)	
Form							
	Oval	14(58,3)	13(54,2)	0,92**	14(58,3)	13(54,2)	0,79**
	Round	0(0,0)	0(0,0)		1(4,2)	0(0,0)	
	Irregular	10(41,7)	11(45,8)		9(37,5)	11(45,8)	
Orientation							
	Parallel	23(95,8)	22(91,7)	0,75*	24(100,0)	22(91,7)	0,71*
	Not parallel	1(4,2)	2(8,3)		0(0,0)	2(8,3)	
Margins							
	Circumscribed	16(66,7)	16(66,7)	0,95**	15(62,5)	15(62,5)	0,98**
	Indistinct	4(16,7)	4(16,7)		5(20,8)	4(16,7)	
	Angled	0(0,0)	1(4,2)		0(0,0)	2(8,3)	
	Microlobulated	1(4,2)	1(4,2)		2(8,3)	1(4,2)	
	Spiked	3(12,5)	2(8,3)		2(8,3)	2(8,3)	
Rear acoustics							
	Absent	19(79,2)	18(75,0)	0,91**	19(79,2)	18(75,0)	0,92**
	Acoustic reinforcement	0(0,0)	1(4,2)		0(0,0)	1(4,2)	
	Shadow	5(20,8)	5(20,8)		5 (20,8)	5(20,8)	

* Kappa;

** Kendall's Tau-b; n - absolute frequency; % - relative Frequency; ABUS - automated breast ultrasound; HHUS - hand held ultrasound; P1 - researcher 1; P2 - researcher 2

Interobserver agreement for automated ultrasonography (ABUS) was almost perfect (for echogenicity, shape, orientation, and margins) to perfect (for posterior acoustic features), being somewhat lower, but still substantial or strong, relative to the BI-RADS^^®^^ classification. For conventional ultrasonography (HHUS) the interobserver agreement was perfect for the variables shape, orientation and posterior acoustic characteristics, almost perfect for echogenicity and margins and substantial or strong for the BI-RADS^®^ classification (Table 3).

**Table 3:** Characterization and agreement analysis of BI-RADS^^®^^ classification of breast lesions and ultrasound descriptors of lesions submitted to biopsy, between researchers, in each method

		ABUS		*Kappa*	HHUS		*Kappa*
		P1	P2		P1	P2	
BI-RADS^^®^^							
	Benign	9(33,3)	13(48,1)	0,63*	9(33,3)	11(40,7)	0,68*
	Suspect	18(66,7)	14(51,9)		18(66,7)	16(59,3)	
Echogenicity							
	Hypoechoic	10(41,7)	10(41,7)	0,85**	9(37,5)	11(45,8)	0,85**
	Isoechoic	0(0,0)	1(4,2)		0(0,0)	0(0,0)	
	Heterogeneous	8(33,3)	8(33,3)		9(37,5)	8(33,3)	
	> 1 pattern	3(12,5)	1(4,2)		3(12,5)	1(4,2)	
	Solid-cystic	3(12,5)	4(16,7)		3(12,5)	4(16,7)	
Form							
	Oval	14(58,3)	14(58,3)	0,91**	13(54,2)	13(54,2)	1,00**
	Round	0(0,0)	1(4,2)		0(0,0)	0(0,0)	
	Irregular	10(41,7)	9(37,5)		11(45,8)	11(45,8)	
Orientation							
	Parallel	23(95,8)	24(100,0)	0,83*	22(91,7)	22(91,7)	1,00*
	Not parallel	1(4,2)	0(0,0)		2(8,3)	2(8,3)	
Margins							
	Circumscribed	16(66,7)	15(62,5)	0,87**	16(66,7)	15(62,5)	0,98**
	Indistinct	4(16,7)	5(20,8)		4(16,7)	4(16,7)	
	Angled	0(0,0)	0(0,0)		1(4,2)	2(8,3)	
	Microlobulated	1(4,2)	2(8,3)		1(4,2)	1(4,2)	
	Spiked	3(12,5)	2(8,3)		2(8,3)	2(8,3)	
Rear acoustics							
	Absent	19(79,2)	19(79,2)	1,00**	18(75,0)	18(75,0)	1,00**
	Acoustic reinforcement	0(0,0)	0(0,0)		1(4,2)	1(4,2)	
	Shadow	5(20,8)	5(20,8)		5(20,8)	5(20,8)	

* Kappa;

** Kendall's Tau-b;

n - absolute frequency; % - relative Frequency; ABUS - automated breast ultrasound; HHUS - hand held ultrasound; P1 - researcher 1; P2 - researcher 2

Through the analysis of the ROC curve of each method evaluated by the two researchers, it was observed that both methods presented good accuracy, with statistically significant p values. The ROC curve area for HHUS was 0.67 (p=0.003) for researcher 1 and 0.72 (p<0.001) for researcher 2. The ROC curve area for ABUS was 0.69 (p=0.001) for researcher 1 and 0.78 (p<0.001) for researcher 2. The indicators’ evaluation for each method was carried out using the Galen and Gambino's method (1975),^(10)^ demonstrating that there are no statistically significant differences between the conventional and automated methods regarding the indicators related to the accuracy of each one (Table 4).

**Table 4:** Accuracy indicators of breast ultrasound according to the method performed (conventional – HHUS or automated – ABUS) in the evaluation of both researchers

Method Indicators	Researcher 1		p-value*	Researcher 2		p-value*
	ABUS (%)	HHUS (%)		ABUS (%)	HHUS (%)	
Sensitivity	100	100	1,0	100	100	1,0
Specificity	38,9	33,3	0,1	55,6	44,4	0,1
Positive predictive value	35,2	33,3	0,5	42,8	37,5	0,6
Negative predictive value	100	100	1,0	100	100	1,0
Accuracy	54,2	50	0,6	66,7	58,3	0,3
False negative rate	0	0	1,0	0	0	1,0
False positive rate	61,1	66,7	0,4	44,4	55,6	0,2

* p= Galen and Gambino;

ABUS - automated breast ultrasound; HHUS - hand held ultrasound

## Discussion

We observed less medical time dedicated to automated breast ultrasound compared to conventional breast ultrasound, maintaining accuracy. The agreement between the methods and interobservers was substantial to perfect, according to each evaluated variables. Thus, in this sample, the echographic evaluation of the breasts with the automated method presented diagnostic possibilities similar to those of the conventional method, maintaining the main findings’ reproducibility.

In the diagnostic context in which our results were obtained, although we still do not have long-term evidence, the studies carried out to date have also shown that the performance of ABUS is similar to that of conventional ultrasound. No statistically significant differences have been observed between the methods in terms of sensitivity and specificity.^(2,3,8,11)^

However, regarding the medical time dedicated to each of the methods, significantly lower time averages have been observed for automated ultrasound in many studies.^(4,12-14)^ The study by Vourtsis and Kachulis (2017)^(13)^ stands out, which evaluated 1886 patients, symptomatic or not, with the automated ultrasound images evaluated by two breast radiologists, obtaining an average of 3 minutes. This average is much lower than the average of 19 minutes of the randomized clinical trial ACRIN 6666, which with 2725 participants, represents the study with the largest sample and the highest level of scientific evidence regarding the evaluation of the time dedicated to the conventional method of performing breast ultrasound.^(5)^

Our average time for the automated method was 7.38 minutes, similar to that of Wilczek et al. (2016),^(12)^ but higher than most studies.^(13-15)^ This result may be related to the period of learning the new technique in which both researchers were inserted. For the conventional method, they had extensive experience, probably interfering with our average time for this method, which was 10.77 minutes. This value is considerably lower than the average observed in the literature,^(5,16,17)^ but similar to that of the study by Tutar et al. (2020).^(18)^ In this cross-sectional study with 340 patients undergoing screening, an average of 12.5 minutes was obtained by physicians breast radiologists.^(18)^

As limitations of our study, we have the small number of patients included, the observational methodological design and the difference in the researchers’ experience time with each of the methods. However, we obtained the minimum number of participants indicated in the sample size calculation and our results were consistent with larger studies. These observations suggest that new evaluations with automated ultrasound in the diagnostic context will certainly be important for the continuation of its use in this scenario.

## Conclusion

We observed that automated breast ultrasound required less time for the medical professional to interpret the images and complete the BI-RADS^^®^^ classification compared to conventional ultrasound. There was substantial or strong to perfect interobserver agreement and substantial or strong to almost perfect agreement between the methods, maintaining accuracy.
